# Dietary supplementation with 0.4% *L*-arginine between days 14 and 30 of gestation enhances NO and polyamine syntheses and water transport in porcine placentae

**DOI:** 10.1186/s40104-022-00794-0

**Published:** 2022-12-08

**Authors:** Cassandra M. Herring, Fuller W. Bazer, Gregory A. Johnson, Heewon Seo, Shengdi Hu, Mohammed Elmetwally, Wenliang He, Daniel B. Long, Guoyao Wu

**Affiliations:** 1 Department of Animal Science, Texas A&M University, College Station, TX 77843 USA; 2 Department of Veterinary Integrative Biosciences, Texas A&M University, College Station, TX 77843 USA

**Keywords:** Angiogenesis, Arginine, Fetus, Placenta, Reproduction

## Abstract

**Background:**

Most embryonic loss in pigs occurs before d 30 of gestation. Dietary supplementation with *L*-arginine (Arg) during early gestation can enhance the survival and development of conceptuses (embryo/fetus and its extra-embryonic membranes) in gilts. However, the underlying mechanisms remain largely unknown.

**Methods:**

Between d 14 and 30 of gestation, each gilt was fed daily 2 kg of a corn- and soybean-meal based diet (12% crude protein) supplemented with either 0.4% Arg (as Arg-HCl) or an isonitrogenous amount of *L*-alanine (Control). There were 10 gilts per treatment group. On d 30 of gestation, gilts were fed either Arg-HCl or *L*-alanine 30 min before they were hysterectomized, followed by the collection of placentae, embryos, fetal membranes, and fetal fluids. Amniotic and allantoic fluids were analyzed for nitrite and nitrate [NOx; stable oxidation products of nitric oxide (NO)], polyamines, and amino acids. Placentae were analyzed for syntheses of NO and polyamines, water and amino acid transport, concentrations of amino acid-related metabolites, and the expression of angiogenic factors and aquaporins (AQPs).

**Results:**

Compared to the control group, Arg supplementation increased (*P* < 0.05) the number of viable fetuses by 1.9 per litter, the number and diameter of placental blood vessels (+ 25.9% and + 17.0% respectively), embryonic survival (+ 18.5%), total placental weight (+ 36.5%), the total weight of viable fetuses (+ 33.5%), fetal crown-to-rump length (+ 4.7%), and total allantoic and amniotic fluid volumes (+ 44.6% and + 75.5% respectively). Compared to control gilts, Arg supplementation increased (*P* < 0.05) placental activities of GTP cyclohydrolase-1 (+ 33.1%) and ornithine decarboxylase (+ 29.3%); placental syntheses of NO (+ 26.2%) and polyamines (+ 28.9%); placental concentrations of NOx (+ 22.5%), tetrahydrobiopterin (+ 21.1%), polyamines (+ 20.4%), cAMP (+ 27.7%), and cGMP (+ 24.7%); total amounts of NOx (+ 61.7% to + 96.8%), polyamines (+ 60.7% to + 88.7%), amino acids (+ 39% to + 118%), glucose (+ 60.5% to + 62.6%), and fructose (+ 41.4% to + 57.0%) in fetal fluids; and the placental transport of water (+ 33.9%), Arg (+ 78.4%), glutamine (+ 89.9%), and glycine (+ 89.6%). Furthermore, Arg supplementation increased (*P* < 0.05) placental mRNA levels for angiogenic factors [*VEGFA120* (+ 117%), *VEGFR1* (+ 445%), *VEGFR2* (+ 373%), *PGF* (+ 197%), and *GCH1* (+ 126%)] and AQPs [*AQP1* (+ 280%), *AQP3* (+ 137%), *AQP5* (+ 172%), *AQP8* (+ 165%), and *AQP9* (+ 127%)].

**Conclusion:**

Supplementing 0.4% Arg to a conventional diet for gilts between d 14 and d 30 of gestation enhanced placental NO and polyamine syntheses, angiogenesis, and water and amino acid transport to improve conceptus development and survival.

## Introduction

Pigs suffer high rates of embryonic mortality, especially during the peri-implantation period of pregnancy [1, 2] when embryos elongate rapidly, signal for pregnancy recognition, and attach to the uterine wall [3, 4]. Maternal nutrition plays an important role in the development and subsequent survival of conceptuses (embryo/fetus and its extra-embryonic membranes), especially the maternal dietary intake of amino acids (AAs) [5–7]. Both low and high dietary protein intake can contribute to problems with fetal development and embryonic death due to deficiencies and excesses of AAs, respectively [8, 9]. Specifically, *L*-arginine (Arg) is a “conditionally essential AA” in the diet that is important for optimal embryonic development and survival by affecting placental growth [10–12]. Arg is the nitrogenous substrate for the synthesis of nitric oxide (NO), which is essential for placental angiogenesis (the sprouting of new blood vessels from existing ones) [13, 14] and the regulation of cell metabolism [15–17]. Angiogenesis provides the physical conduit for utero-placental blood flow to enhance mother to fetus exchanges of water, AAs, and other nutrients, as well as gases and wastes [11]. Arg is also known to be a precursor for syntheses of polyamines, ornithine, creatine, agmatine, and homoarginine, each of which has enormous physiological significance [18–21]. These biologically important substances are essential for conceptus growth and survival [22]. In support of this view, we found that dietary supplementation with 0.4% or 0.8% Arg to gilts between d 14 and 25 of gestation enhanced placental syntheses of NO and polyamines, as well as the placental expression of angiogenic factors [15].

Previous studies demonstrated that dietary supplementation with Arg to gilts during specific periods of gestation improved the placental expression of antioxidative genes [23], embryonic and fetal survival rates, as well as conceptus growth [5, 6, 24–26]. For example, Mateo et al. [26] reported that supplementation of 0.83% Arg between d 30 and d 114 of gestation increased litter size in gilts by 2. In addition, Li et al. [24] found that dietary supplementation with 0.4% and 0.8% Arg between d 14 and d 25 of gestation increased the litter size in gilts by 2 and amniotic fluid volume. Similar results were reported for rats [27, 28]. Interestingly, dietary supplementation with 0.8% Arg from d 0 to d 25 impaired embryonic survival due to reductions in the number of corpora luteum (CL) and concentrations of progesterone in the maternal plasma because of excessive NO production [29], indicating the importance of the timing of maternal Arg provision.

Water is transported rapidly across the placenta and accumulates in allantoic and amniotic fluids during early gestation to support conceptus growth, development, and survival in mammals, including swine [30, 31]. Aquaporins (AQPs) are plasma membrane proteins that allow rapid water transport across membranes [32] and are also essential for placental development [33]. AQPs are activated by multiple signaling pathways, including cGMP, cAMP, mitogen-activated protein kinases, protein kinase C, and phosphatidylinositide 3-kinases/protein kinase B/mechanistic target of rapamycin [34–36]. To date, 13 isoforms of AQP have been discovered in mammals [32], 12 of which are expressed in the female reproductive tract [37]. Pigs can potentially use AQP1, AQP5, AQP8, and AQP9 to transport water from the endometrial bloodstream to the allantoic bloodstream or allantoic fluid [38].

Although the timing and dose of Arg supplementation to pregnant gilts has been studied, the exact mechanism through which it increases embryonic survival is not fully understood [37, 39]. We conducted this study to test the hypothesis that supplementation of 0.4% Arg to gilts between d 14 and d 30 of gestation would increase embryonic survival and development by increasing the placental expression of angiogenic factors and AQP. We extended the period of Arg supplementation to d 30 of gestation, so that the placentae of gilts could be successfully mounted into Ussing chambers to determine water and AA transport.

## Materials and methods

### Experimental design

Twenty gilts (F1 crosses of Yorkshire × Landrace sows and Duroc × Hampshire boars) with a body weight of 100–125 kg were bred at the onset of the second estrus and 12 h later. The day of breeding was recorded as d 0 of gestation. Following breeding, gilts were assigned randomly to 1 of 2 treatment groups, 0 (control) or 0.4% Arg (as 0.484% Arg-HCl; Ajinomoto Co., Inc., Tokyo, Japan), with 10 gilts in each treatment group. An isonitrogenous amount of 0.83% of *L*-alanine (Ajinomoto Co., Inc., Tokyo, Japan) and 0.43% cornstarch were added to the 0 and 0.4% Arg diets, respectively, as top dressing. Each gilt was fed 1 kg of a corn- and soybean meal-based diet containing 12% crude protein twice daily (0700 and 1800 h) beginning on d 0 of gestation, with a feed intake of 2 kg/d [24]. The content of nutrients in the basal diet was the same as reported in our previous study [29], which contained 12.0% crude protein (including 0.70% Arg and 0.57% lysine, as analyzed by high-performance liquid chromatography (HPLC) following acid hydrolysis [40]) and 12.9 MJ metabolizable energy/kg. Either 0.4% Arg or the isonitrogenous amount of *L*-alanine was supplemented to the basal diet between d 14 and d 30 of gestation.

### Hysterectomy and tissue collection

On d 30 of gestation, gilts were fed either Arg-HCl or *L*-alanine, and hysterectomized and necropsied within 30 min of consuming the supplement. Gilts were anesthetized with an intramuscular injection of 10 mg Telazol (Midwest Veterinary Supply, Lakeville, MN, USA) per kg of body weight that was followed by the inhalation of 1%–5% isofluorane to achieve a surgical plane of anesthesia [24]. Blood was collected from the uterine vein and artery before euthanasia, and after euthanasia gilts were hysterectomized to obtain uteri and conceptuses. Euthanasia was performed with an intracardiac injection of saturated KCl. The number of CL, the number of live fetuses, placental weight, fetal body weight, fetal crown-to-rump length (the distance from the crown of the head to the base of the tail), the volumes of amniotic and allantoic fluid in viable conceptuses, and the number and diameter of placental blood vessels were determined, as we described previously [22, 41]. Briefly, a picture was taken of each placenta. Three placentae (from the first, middle, and last fetuses within the left uterine horn) and three placentae (from the first, middle, and last fetuses within the right uterine horn) from each gilt were evaluated to determine the total number of blood vessels per 1 cm^2^, and to measure the diameter of the central blood vessel under a microscope (40 × objective). For each variable, the mean of the six placental measurements was calculated to represent the value for the gilt. Samples of placentae were snap-frozen in liquid nitrogen. For the analyses of metabolites, allantoic or amniotic fluid from each viable conceptus of the same gilt was combined in equal proportions.

### Determination of NO and polyamine syntheses by placentae

Placental tissues (~ 200 mg) were rinsed three times with 1 mL of oxygenated (95% O_2_/5% CO_2_; v/v) custom-made Dulbecco's-modified Eagle medium containing physiological concentrations of AAs (including 0.2 mmol/L Arg), 5 mmol/L *D*-glucose, 100 units/mL penicillin, 100 µg/mL streptomycin and 0.25 µg/mL amphotericin B [42], preincubated at 37 °C for 0.5 h in 4 mL of fresh oxygenated medium, and then incubated at 37 °C for 6 h in 1 mL of fresh oxygenated medium that contained 5 mmol/L *D*-glucose, 0.2 mmol/L Arg, and concentrations of other AAs found in the plasma of gilts [42]. At the end of a 6-h incubation period, the medium was analyzed for nitrite plus nitrate (stable oxidation products of NO). In all experiments, the medium incubated without cells was analyzed as the blank. Nitrite and nitrate in culture medium were quantified by HPLC as we previously described [43, 44].

To determine effects of treatment on polyamine synthesis, placentae were incubated as described above, except that the medium contained 0.5 mmol/L *L*-[1-^14^C]ornithine [45]. ^14^C-labeled putrescine, spermidine, and spermine were separated by HPLC and their radioactivities were measured by a liquid scintillation counter, as we described previously [46, 47]. The rates of the production of putrescine, spermidine, and spermine were calculated on the basis of their radioactivities and the specific radioactivity of *L*-[1-^14^C]ornithine in the incubation medium.

### Determination of NOx, polyamines, BH_4_, cAMP, cGMP, glucose, and fructose

For the analysis of NOx (nitrite and nitrate) and polyamines (the sum of putrescine, spermidine and spermine), placentae (~ 50 mg) were homogenized in 1 mL of 1.5 mol/L HClO_4_, followed by neutralization with 0.5 mL of 2 mol/L K_2_CO_3_ [45]. NOx (nitrite plus nitrate) and polyamines were determined using our established HPLC methods [43, 44]. For BH_4_ analysis, tissues (~ 50 mg) were homogenized in 0.5 mL of 0.1 mol/L phosphoric acid containing 5 mmol/L dithioerythritol and 60 µL of 2 mol/L trichloacetic acid, and the tissue extract was used for BH_4_ analysis by HPLC [13]. For the determination of cGMP in placentae, the tissue (~ 100 mg) was homogenized in 1 mL of 1.5 mol/L HClO_4_, followed by the neutralization with 0.5 mL of 2 mol/L K_2_CO_3_. The extract was analyzed for 3’-5’-cGMP using the cGMP Enzymeimmunoassay Biotrak System (GE Healthcare, Chalfont St Giles, Buckinghamshire, UK). cAMP was analyzed by an HPLC method involving the precolumn derivatization with 2-chloroacetaldehyde and fluorescence detection, as we previously described [48]. Glucose, fructose, and glycerol were determined as described by He et al. [49], Li et al. [24], and Jobgen et al. [50], respectively.

### Determination of enzymatic activities

Fresh placental tissue (~ 100 mg) was used to prepare the cytosolic fraction for the assay of ornithine decarboxylase (ODC) activity with the use of 2 mmol/L *L*-[1-^14^C]ornithine (2500 dpm/nmol), as we described previously [45]. The activities of constitutive NO synthase (cNOS), and inducible NO synthase (iNOS) in frozen placental tissue were measured using *L*-[U-^14^C]Arg [13]. The activity of GTP-cyclohydrolase-1 [GTP-CH1; the key enzyme for BH_4_ synthesis) in frozen placental tissue was determined using desalted tissue extract and 2 mmol/L GTP, as we described previously [51, 52].

### Determination of placental transport of water and AAs

Transport of ^3^H_2_O was measured with the use of Ussing chambers (Physiologic Instruments, San Diego, CA, USA) containing 5 mL of oxygenated (95% O_2_/5% CO_2_) Krebs buffer as well as physiological concentrations of AAs and glucose, as we described [53, 54]. Pieces of placental tissue (1 cm^2^) were mounted onto Ussing chambers, followed by the addition of ^3^H_2_O (20 µL), 0.2 mmol/L *L*-[U-^14^C]Arg, 0.5 mmol/L *L*-[U-^14^C]glutamine, or 1 mmol/L [U-^14^C]glycine (similar to physiological concentrations of AAs in the pig plasma) to the “mucosal” side of each chamber. The specific radioactivity of ^3^H_2_O on the “mucosal” side of the chamber was 500 dpm/µL H_2_O, whereas the specific radioactivities of 0.2 mmol/L *L*-[U-^14^C]Arg, 0.5 mmol/L *L*-[U-^14^C]glutamine, and 1 mmol/L [U-^14^C]glycine on the “mucosal” side of the chamber were 3 × 10^4^, 1.2 × 10^4^, and 6 × 10^3^ dpm/nmol, respectively. Thereafter, an aliquot of 20 µL solution was obtained from the “serosal” side of the chamber at 5, 10 and 15 min for the measurement of ^3^H_2_O and ^14^C radioactivity using a Packard liquid scintillation counter [47].

### RNA extraction, reverse transcription and quantitative PCR

Placental tissue (~ 100 mg) was homogenized with 1 mL of TRIzol (Invitrogen, Waltham, MA, USA) and RNA was extracted with chloroform and precipitated with isopropanol [55–57]. RNA was washed with 75% ethanol. Total RNA was measured using a NanoDrop ND 1000 spectrophotometer. cDNA was synthesized using the SuperScript First Strand Synthesis System for RT-PCR (Invitrogen). RT-qPCR was performed using the SYBR Green and the Applied Biosystems 7900HT Real Time PCR system [56]. Sequences of primers, which were designed using the Primer-BLAST software (http://www.ncbi.nlm.nih.gov/tools/primer-blast/) for the quantitative RT-PCR analysis, are shown in Table 1. Tubulin α 1b (*TUBA1B*) was used as the housekeeping gene [55]. The relative expression values were calculated using the ΔΔCt method [57].

**Table 1:** Sequences of primers used for the quantitative RT-PCR analysis of genes in porcine placentae

Gene	Primer sequence	Accession number
*NOS3*	Forward: 5’- ATCTTCAGCCCCAAACGGAG -3’	NM_214295.1
	Reverse: 5’- TTTCCACCGAGAGGACCGTA -3’	
*VEGF120*	Forward: 5’- AAGGCCAGCACATAGGAGAG -3’	KJ729036
	Reverse: 5’- CCTCGGCTTGTCACATTTTT-3’	
*VEGF164*	Forward: 5’- GAGGCAAGAAAATCCCTGTG -3’	NM214084
	Reverse: 5’- TCACATCTGCAAGTACGTTCG- 3’	
*VEGFR1*	Forward: 5’- CACCCCGGAAATCTATCAGATC -3’	EU714325.1
	Reverse: 5’- GAGTACGTGAAGCCGCTGTTG -3’	
*VEGFR2*	Forward: 5’- GAAATGGCTTCATCCTCCAA -3’	AF513909.1
	Reverse: 5’- CAAGGAAGACTTGGCTCAGG -3’	
*GCH1*	Forward: 5’- AGTTCTTGGCCTCAGCAAAC -3’	XM_021102249.1
	Reverse: 5’ TGCTTCAACCACTACTCCGAC -3’	
*PGF*	Forward: 5’- CATCGTGTCTGTGTACCCCA -3’	FJ177137.1
	Reverse: 5’- TGACATTGACCGTCTCCACG -3’	
*FGF2*	Forward: 5’- GTGCAAACCGTTACCTTGCT -3’	NM_001001855.2
	Reverse: 5’- ACTGCCCAGTTCGTTTCAGT -3’	
*AQP1*	Forward: 5’- TTGGGCTGAGCATTGCCACGC -3’	XM_021078524.1
	Reverse: 5’- CAGCGAGTTCAGGCCAAGGGAGTT -3’	
*AQP2*	Forward: 5’- TCAACCCTGCCGTGACTGTAG -3’	EU636238.1
	Reverse: 5’- GTTGTTGCTGAGGGCATTGAC -3’	
*AQP3*	Forward: 5’- ACCCTTATCCTCGTGATGTTT -3’	HQ888860.1
	Reverse: 5’- CATTCGCATCTACTCCTTGTG -3’	
*AQP4*	Forward: 5’- TCTGGCTATGCTTATCTTTGTCC -3’	NM_001110423.1
	Reverse: 5’- CGATGCTAATCTTCCTGGTGC -3’	
*AQP5*	Forward: 5’- TGAGTCCGAGGAGGATTGGG -3’	NM_001110424.1
	Reverse: 5’- GAGGCTTCGCTGTCATCTGTTT -3’	
*AQP8*	Forward: 5’- GGTGCCATCAACAAGAAGACG -3’	EU220426.1
	Reverse: 5’- CCGATAAAGAACCTGATGAGCC -3’	
*AQP9*	Forward: 5’- TTTGCTGATGGAAAACTGCTC -3’	NM_001112684.1
	Reverse: 5’- CTCTGGTTTGTCCTCCGATTGT -3’	
*AQP10*	Forward: 5’-TGGGCGTTATACTAGCCATCTAC-3’	EU582021
	Reverse: 5’-GGTTGGGCACAGTTTACTTCCT-3’	
*AQP11*	Forward: 5’- CGTCTTGGAGTTTCTGGCTACC -3’	EU220425
	Reverse: 5’- CCTGTCCCTGACGTGATACTTG -3’	
*TUBA1B*	Forward: 5’- GCTGCCAATAACTATGCCCG-3’	NM_001044544
	Reverse: 5’- ACCAAGAAGCCCTGAAGACC-3	

### Statistical analysis

All data, except for embryonic survival rates, were analyzed statistically using the unpaired *t*-test [58]. Embryonic survival rates were compared using the *X*^*2*^ analysis [58]. Probability values ≤ 0.05 were considered statistically significant.

## Results

### Reproductive performance of gilts

After dietary supplementation with 0 (control) or 0.4% Arg between d 14 and 30 of gestation, gilts were euthanized and hysterectomized to assess their reproductive performance. Maternal body weight and CL number did not differ (*P* > 0.05) between control and Arg-supplemented gilts (Table 2). Embryonic survival rates were determined as the number of live fetuses being divided by the number of CL present on the ovaries at the time of necropsy. Compared with the control group, dietary supplementation with 0.4% Arg enhanced (*P* < 0.05) the number of viable fetuses by 1.9 per litter. The embryonic survival rate of the Arg-supplemented gilts was 18.5% greater (*P* < 0.05) than that of the control group (*P* < 0.05) (Table 2). Compared to control gilts, Arg supplementation increased (*P* < 0.05) total placental weight (36.5%), the total weight of viable fetuses (33.5%), fetal crown-to-rump length (4.7%), total allantoic fluid volume (44.6%), and total amniotic fluid volume (75.5%) (Table 2).

**Table 2:** Reproductive performance and placental angiogenesis of gilts fed diets supplemented with 0 (control) or 0.4% *L*-arginine (Arg) between d 14 and d 30 of gestation

Variable	Control	0.4% Arg
Maternal body weight at breeding, kg	119.7 ± 2.6	118.1 ± 3.4
Number of corpora lutea/littler, n	14.1 ± 0.6	13.9 ± 0.7
Number of live fetuses/litter, n	11.2 ± 0.7	13.1 ± 0.4^†^
Embryonic survival rate, %	79.5 ± 3.5	94.2 ± 2.1^†^
Weight of the viable fetus, g	1.75 ± 0.06	1.98 ± 0.08^†^
Weight of total viable fetuses/litter, g	19.4 ± 0.9	25.9 ± 1.1^*^
Fetal crown-to-rump length, mm	25.4 ± 0.35	26.6 ± 0.43^†^
Weight of the placenta for the live fetus, g	31.6 ± 1.1	36.8 ± 1.7^†^
Weight of total placentae/litter, g	353 ± 22	482 ± 26^*^
Volume of allantoic fluid/viable fetus, mL	185 ± 11	229 ± 15^†^
Total volume of allantoic fluid/litter, mL	2,075 ± 179	3,001 ± 262^*^
Volume of amniotic fluid/viable fetus, mL	1.31 ± 0.04	1.97 ± 0.17^*^
Total volume of amniotic fluid/litter, mL	14.7 ± 0.8	25.8 ± 2.5^*^
Number of placental blood vessels, n/cm^2^	9.53 ± 0.40	12.0 ± 0.95^†^
Diameter of placental blood vessels, mm	7.18 ± 0.25	8.40 ± 0.32^*^

Data are mean values ± SEM, *n *= 10. Embryonic survival rate was calculated as number of live fetuses per number of corpora lutea present on the ovaries at the time of necropsy on d 30 of gestation

^†^*P* < 0.05 vs the control group

^*^P < 0.01 vs the control group

### Effects of dietary Arg supplementation on the concentrations of AAs and related metabolites in the maternal plasma and placenta, as well as their total amounts in fetal fluids of gilts

Compared to control gilts, Arg supplementation increased (*P* < 0.05) concentrations of Arg, ornithine, and proline in maternal plasma (37.2%, 29.6%, and 16.4%, respectively) and placentae (14.4%, 11.7%, and 15.7%, respectively), but had no effect (*P* > 0.05) on those of other AAs (Table 3). Concentrations of glucose, fructose, and glycerol in the maternal plasma and placentae did not differ (*P* > 0.05) between the control and Arg groups of gilts. By contrast, concentrations of alanine in the maternal plasma and placentae were 98.4% and 11.1% greater (*P* < 0.05) in control gilts than in Arg-supplemented gilts. Compared to the control group, Arg supplementation increased (*P* < 0.05) concentrations of Arg (50.3%), glutamine (12.5%), glycine (33.3%), ornithine (25.7%), and serine (27.1%) in allantoic fluid, but had no effect (*P* > 0.05) on those of other AAs, glucose and fructose (Table 3). Concentrations of all measured AAs, glucose and fructose in amniotic fluid did not differ (*P* > 0.05) between the control and Arg-supplemented gilts. Dietary supplementation with Arg reduced (*P* < 0.01) concentrations of glycerol in allantoic and amniotic fluids by 27.0% and 30.1% respectively, compared with control gilts. Due to increased volumes of allantoic and amniotic fluids, total amounts of all AAs, glucose and fructose in the fetal fluids were 31%–117.8%, 60.5%–62.6%, and 41.4%–57.0% greater (*P* < 0.01) respectively in Arg-supplemented than in control gilts. Total amounts of glycerol in these fluids did not differ (*P* > 0.05) between the two groups of gilts (Table 4).

**Table 3:** Effects of dietary supplementation with 0 (control) or 0.4% *L*-arginine (Arg) between d 14 and d 30 of gestation on concentrations of amino acids and related metabolites in the maternal uterine arterial plasma and in fetal fluids of gilts

Variable	Maternal uterine plasma		Placenta		Allantoic fluid		Amniotic fluid	
	Control	0.4% Arg	Control	0.4% Arg	Control	0.4% Arg	Control	0.4% Arg
Ala	843 ± 55	425 ± 20^*^	661 ± 20	595 ± 23^†^	258 ± 16	268 ± 18	269 ± 15	254 ± 13
β-Alanine	7.2 ± 0.46	7.1 ± 0.39	16.3 ± 0.8	15.8 ± 1.1	9.4 ± 0.68	9.7 ± 0.84	8.1 ± 0.55	8.2 ± 0.42
Arg	148 ± 6.5	203 ± 7.9^*^	367 ± 12	420 ± 15^†^	181 ± 7.4	272 ± 9.4^*^	152 ± 10	155 ± 9.1
Asn	67.2 ± 4.4	62.0 ± 3.7	164 ± 7.6	157 ± 8.1	62.2 ± 3.0	66.7 ± 3.5	52.0 ± 2.2	53.3 ± 2.6
Asp	14.1 ± 0.7	12.6 ± 0.9	353 ± 16	346 ± 18	15.5 ± 0.65	16.9 ± 0.72	15.2 ± 0.71	15.6 ± 0.52
Citrulline	63.5 ± 2.6	62.3 ± 2.3	19.7 ± 1.0	19.3 ± 1.1	10.3 ± 0.54	10.6 ± 0.47	8.2 ± 0.46	8.5 ± 0.39
Cys^1^	196 ± 7.8	204 ± 9.3	266 ± 9.4	269 ± 10	76.7 ± 3.7	78.0 ± 4.4	35.7 ± 1.8	35.3 ± 1.7
Glu	203 ± 9.2	178 ± 12	705 ± 22	657 ± 19	129 ± 5.9	143 ± 6.2	132 ± 7.4	125 ± 8.1
Gln	472 ± 20	459 ± 18	816 ± 25	805 ± 32	630 ± 23	709 ± 27^†^	680 ± 18	692 ± 35
Gly	779 ± 34	738 ± 41	873 ± 40	883 ± 30	847 ± 38	1,129 ± 57^*^	347 ± 11	339 ± 14
His	78.7 ± 4.9	80.1 ± 3.8	185 ± 7.3	173 ± 8.0	78.8 ± 5.4	82.1 ± 5.7	48.6 ± 3.0	49.4 ± 2.4
Hyp	21.9 ± 1.6	21.4 ± 1.8	102 ± 5.9	108 ± 7.4	51.6 ± 2.4	52.8 ± 3.0	35.2 ± 1.6	36.4 ± 2.0
Ile	121 ± 5.2	114 ± 6.5	177 ± 6.9	171 ± 5.8	29.8 ± 1.5	29.1 ± 2.3	51.5 ± 2.7	50.8 ± 3.2
Leu	207 ± 8.0	189 ± 7.2	225 ± 11	216 ± 8.9	65.2 ± 4.2	66.3 ± 3.4	133 ± 8.4	139 ± 7.0
Lys	176 ± 16	168 ± 13	410 ± 19	414 ± 16	358 ± 15	371 ± 17	204 ± 5.9	198 ± 8.3
Met	48.8 ± 2.4	46.3 ± 1.7	195 ± 9.0	174 ± 9.8	21.6 ± 1.6	21.3 ± 1.1	48.1 ± 2.0	47.4 ± 3.7
Ornithine	81.0 ± 6.6	105 ± 7.6^†^	163 ± 5.4	182 ± 6.0^†^	113 ± 6.1	142 ± 6.5^†^	104 ± 6.0	102 ± 6.7
Phe	75.6 ± 3.8	70.3 ± 4.2	175 ± 5.4	167 ± 6.9	37.7 ± 1.9	38.4 ± 2.6	70.5 ± 4.4	71.2 ± 3.7
Pro	238 ± 7.6	277 ± 9.5^*^	383 ± 13	443 ± 16^*^	256 ± 13	277 ± 8.5	104 ± 6.5	108 ± 8.6
Ser	124 ± 5.8	109 ± 6.5	486 ± 15	461 ± 19	569 ± 26	723 ± 28^*^	365 ± 17	361 ± 15
Taurine	107 ± 7.5	114 ± 6.1	936 ± 37	963 ± 35	461 ± 20	458 ± 22	114 ± 7.5	110 ± 6.2
Thr	175 ± 8.2	166 ± 9.5	390 ± 13	376 ± 11	221 ± 11	215 ± 13	229 ± 12	224 ± 16
Trp	56.8 ± 1.9	55.4 ± 2.3	68.5 ± 3.7	73.4 ± 3.4	14.1 ± 1.0	14.5 ± 1.1	14.3 ± 0.86	13.6 ± 0.94
Tyr	97.5 ± 6.2	96.1 ± 7.5	198 ± 7.4	187 ± 8.1	46.9 ± 2.3	48.2 ± 2.6	53.5 ± 3.1	52.8 ± 2.9
Val	267 ± 10	254 ± 13	286 ± 8.8	108 ± 7.4	88.6 ± 6.1	90.7 ± 5.8	175 ± 8.8	169 ± 9.1
Glucose	5,341 ± 83	5,279 ± 74	294 ± 15	286 ± 17	1,394 ± 84	1,540 ± 61	1,496 ± 75	1,415 ± 64
Fructose	505 ± 31	508 ± 38	78.0 ± 3.2	80.5 ± 3.7	2,538 ± 169	2,467 ± 121	2,515 ± 107	2,305 ± 101
Glycerol	115 ± 6.3	121 ± 7.4	59.2 ± 3.4	61.3 ± 3.9	230 ± 9.5	168 ± 8.1^*^	116 ± 9.4	81.1 ± 5.3^*^

Values, expressed as nmol/mL for plasma and fetal fluid and as nmol/g tissue for placentae, are means ± SEM, *n* = 10 gilts/treatment group

^1^Cysteine + ½ cysteine; *Hyp*: 4-hydroxyproline

^†^*P* < 0.05 vs the corresponding control group

^*^P < 0.01 vs the corresponding control group

**Table 4:** Effects of dietary supplementation with 0 (control) or 0.4% *L*-arginine (Arg) between d 14 and d 30 of gestation on total amounts of amino acids and related metabolites in fetal fluids of gilts

Variable	Allantoic fluid			Amniotic fluid		
	Control	0.4% Arg	% increase	Control	0.4% Arg	% increase
Ala	537 ± 59	820 ± 110^*^	52.7	3.95 ± 0.33	6.34 ± 0.43^*^	60.5
β-Ala	18.6 ± 0.83	27.4 ± 0.81^*^	47.3	0.12 ± 0.01	0.21 ± 0.02^*^	75.0
Arg	376 ± 36	819 ± 76^*^	117.8	2.20 ± 0.14	3.81 ± 0.19^*^	73.2
Asn	126 ± 8.7	194 ± 12^*^	54.0	0.76 ± 0.05	1.38 ± 0.15^*^	81.6
Asp	32.2 ± 3.0	50.4 ± 4.8^*^	56.5	0.22 ± 0.02	0.40 ± 0.04^*^	81.8
Cit	21.3 ± 2.1	31.5 ± 2.8^*^	47.9	0.12 ± 0.01	0.22 ± 0.03^*^	83.3
Cys^1^	157 ± 13	232 ± 22^*^	47.8	0.53 ± 0.05	0.92 ± 0.11^*^	73.6
Glu	266 ± 24	429 ± 41^*^	61.3	1.91 ± 0.12	3.24 ± 0.40^*^	69.6
Gln	1307 ± 129	2,129 ± 211^*^	62.9	10.0 ± 0.68	17.2 ± 1.1^*^	72.0
Gly	1720 ± 118	3,403 ± 359^*^	97.8	5.02 ± 0.19	8.59 ± 0.80^*^	71.1
His	160 ± 15	246 ± 27^*^	53.8	0.70 ± 0.04	1.30 ± 0.18^*^	85.7
Hyp	105 ± 7.5	156 ± 13^*^	48.6	0.52 ± 0.04	0.91 ± 0.06^*^	75.0
Ile	60.5 ± 4.3	84.1 ± 6.6^*^	39.0	0.76 ± 0.07	1.32 ± 0.17^*^	73.7
Leu	130 ± 6.9	194 ± 12^*^	49.2	1.97 ± 0.18	3.52 ± 0.33^*^	78.7
Lys	735 ± 59	1,109 ± 109^*^	50.9	2.98 ± 0.17	4.99 ± 0.38^*^	67.4
Met	43.6 ± 3.5	62.0 ± 3.7^*^	42.2	0.70 ± 0.03	1.16 ± 0.08^*^	65.7
Ornithine	231 ± 20	418 ± 29^*^	81.0	1.51 ± 0.09	2.54 ± 0.18^*^	68.2
Phe	78.1 ± 8.0	113 ± 9.9^*^	44.7	1.03 ± 0.07	1.79 ± 0.14^*^	73.8
Pro	529 ± 49	837 ± 87^*^	58.2	1.54 ± 0.16	2.72 ± 0.26^*^	76.6
Ser	1175 ± 108	2,137 ± 161^*^	81.9	5.25 ± 0.19	9.34 ± 1.1^*^	77.9
Taurine	945 ± 75	1,353 ± 104^*^	43.2	1.65 ± 0.11	2.70 ± 0.15^*^	63.6
Thr	450 ± 35	633 ± 50^*^	40.7	3.31 ± 0.19	5.56 ± 0.43^*^	68.0
Trp	28.7 ± 2.2	41.6 ± 2.5^*^	44.9	0.21 ± 0.02	0.35 ± 0.04^*^	66.7
Tyr	94.8 ± 5.7	141 ± 10^*^	48.7	0.77 ± 0.05	1.31 ± 0.09^*^	70.1
Val	176 ± 8.7	263 ± 17^*^	49.4	2.58 ± 0.23	4.33 ± 0.45^*^	67.8
Glucose	2866 ± 265	4,600 ± 413^*^	60.5	21.9 ± 1.6	35.6 ± 2.7^*^	62.6
Fructose	5120 ± 338	7,238 ± 505^*^	41.4	37.2 ± 3.0	58.4 ± 4.9^*^	57.0
Glycerol	473 ± 41	506 ± 55	NC	1.72 ± 0.19	2.08 ± 0.21	NC

Values, expressed as µmol/litter, are means ± SEM, *n *= 10 gilts/treatment group

^1^Cysteine + ½ cysteine; *Hyp*: 4-hydroxyproline; *NC*: no change

^†^*P* < 0.05 vs. the corresponding control group

^*^P < 0.01 vs. the corresponding control group

### Effects of dietary Arg supplementation on the concentrations and total amounts of NOx and polyamines in fetal allantoic and amniotic fluids

Concentrations of NOx and polyamines in allantoic and amniotic fluids did not differ (*P* > 0.05) between the control and Arg groups of gilts. Compared with the control group, dietary supplementation with 0.4% Arg increased (*P* < 0.01) total amounts of NOx (61.7%–96.8%) and polyamines (60.7%–88.7%) in allantoic and amniotic fluids of gilt (Table 5) due to their increased volumes. Total amounts of the individual polyamines (putrescine, spermidine, and spermine) in allantoic and amniotic fluids also increased (*P* < 0.01) in response to dietary Arg supplementation.

**Table 5:** Effects of dietary supplementation with 0 (control) or 0.4% *L*-arginine (Arg) between d 14 and d 30 of gestation on the concentrations and total amounts of NOx and polyamines in allantoic and amniotic fluids of gilts

Variable	Allantoic fluid		Amniotic fluid	
	Control	0.4% Arg	Control	0.4% Arg
Concentrations, nmol/mL				
NOx	69.1 ± 2.4	78.0 ± 3.0	45.6 ± 1.8	52.8 ± 2.5
Putrescine	1.64 ± 0.08	1.84 ± 0.14	0.44 ± 0.04	0.48 ± 0.05
Spermidine	2.32 ± 0.15	2.57 ± 0.15	0.71 ± 0.05	0.80 ± 0.06
Spermine	2.54 ± 0.18	2.77 ± 0.16	0.74 ± 0.07	0.85 ± 0.06
Total polyamines	6.50 ± 0.41	7.18 ± 0.43	1.89 ± 0.14	2.12 ± 0.15
Total amounts per litter				
NOx, µmol	141 ± 9.5	228 ± 12^*^	664 ± 37	1307 ± 70^*^
Putrescine, nmol	3,308 ± 195	5,376 ± 478^*^	6.41 ± 0.77	11.4 ± 0.54^*^
Spermidine, nmol	4,685 ± 345	7,536 ± 543^*^	10.3 ± 0.74	19.6 ± 1.5^*^
Spermine, nmol	5,114 ± 409	8,145 ± 614^*^	10.8 ± 1.1	20.9 ± 1.7^*^
Total polyamines, nmol	13,106 ± 929	21,056 ± 1,568^*^	27.5 ± 2.4	51.9 ± 3.1^*^

Data are means ± SEM, *n* = 10 gilts/treatment group. NOx: oxidation end products (nitrite plus nitrate) of NO; total polyamines: putrescine + spermidine + spermine

^*^P < 0.01 vs. the corresponding control group

### Effects of dietary Arg supplementation on the concentrations of metabolites (NOx, polyamines, BH_4_, cAMP, and cGMP), NO and polyamine syntheses, and enzyme activities in the placentae of gilts

The data on the effects of dietary Arg supplementation on concentrations of NOx, polyamines, and BH_4_, NO and polyamine syntheses, and the activities of related enzymes in porcine placentae are summarized in Table 6. Compared with control gilts, dietary supplementation with 0.4% Arg increased (*P* < 0.05) placental concentrations of NOx (22.5%), polyamines (putrescine + spermidine + spermine; 20.4%), BH_4_ (21.1%), cAMP (27.7%), and cGMP (24.7%). The rates of placental syntheses of NO and polyamines were 26.2% and 28.9% greater (*P* < 0.01), respectively, in Arg-supplemented gilts than in the control group. The concentrations and rates of syntheses of the individual polyamines (putrescine, spermidine, and spermine) in placentae also increased (*P* < 0.01) in response to dietary Arg supplementation. Compared with the control group, dietary supplementation with 0.4% Arg increased (*P* < 0.05) the enzymatic activities of GTP-CH1 (33.1%) and ODC (29.3%) in placentae but did not affect (*P* > 0.05) those of cNOS and iNOS.

**Table 6:** Effects of dietary supplementation with 0 (control) or 0.4% *L*-arginine (Arg) between d 14 and d 30 of gestation on the concentrations of NOx and polyamines, NO and polyamine syntheses, and enzyme activities in the placentae of gilts

Variable	Control	0.4% Arg
Placental concentrations		
NOx, nmol/g tissue	46.6 ± 2.5	57.1 ± 2.9^†^
Putrescine, nmol/g tissue	49.1 ± 2.0	60.5 ± 1.8^*^
Spermidine, nmol/g tissue	99.4 ± 4.1	115 ± 3.0^*^
Spermine, nmol/g tissue	101 ± 4.6	125 ± 4.1^*^
Total polyamines, nmol/g tissue	250 ± 10	301 ± 8.2^*^
BH_4_, pmol/g tissue	346 ± 15	419 ± 18^*^
cAMP, pmol/g tissue	202 ± 9.4	258 ± 11^*^
cGMP, pmol/g tissue	15.4 ± 0.8	19.2 ± 0.9^*^
Placental synthesis		
NO, nmol/g tissue/h	12.2 ± 0.48	15.4 ± 0.68^*^
Putrescine, nmol/g tissue/h	0.58 ± 0.02	0.71 ± 0.03^*^
Spermidine, nmol/g tissue/h	0.92 ± 0.05	1.16 ± 0.06^*^
Spermine, nmol/g tissue/h	0.91 ± 0.06	1.26 ± 0.07^*^
Total polyamines, nmol/g tissue/h	2.42 ± 0.11	3.12 ± 0.13^*^
Placental enzyme activity		
cNOS, nmol/g tissue/h	1.30 ± 0.08	1.42 ± 0.09
iNOS, nmol/g tissue/h	0.43 ± 0.02	0.45 ± 0.03
GTP-CH1, nmol/g tissue/h	1.57 ± 0.10	2.09 ± 0.14^*^
ODC, nmol/g tissue/h	8.66 ± 0.57	11.2 ± 0.80^†^

Data are means ± SEM, *n* = 10 gilts/treatment group. *cNOS*: constitutive NO synthase; *GTP-CH1*: GTP cyclohydrolase-I; *iNOS*: inducible NO synthase; *NOx*: oxidation end products (nitrite plus nitrate) of NO; *ODC*: ornithine decarboxylase; total polyamines: putrescine + spermidine + spermine

^†^*P* < 0.05 vs. the control group

^*^P < 0.01 vs. the control group

### Placental angiogenesis

**Fig. 1:**
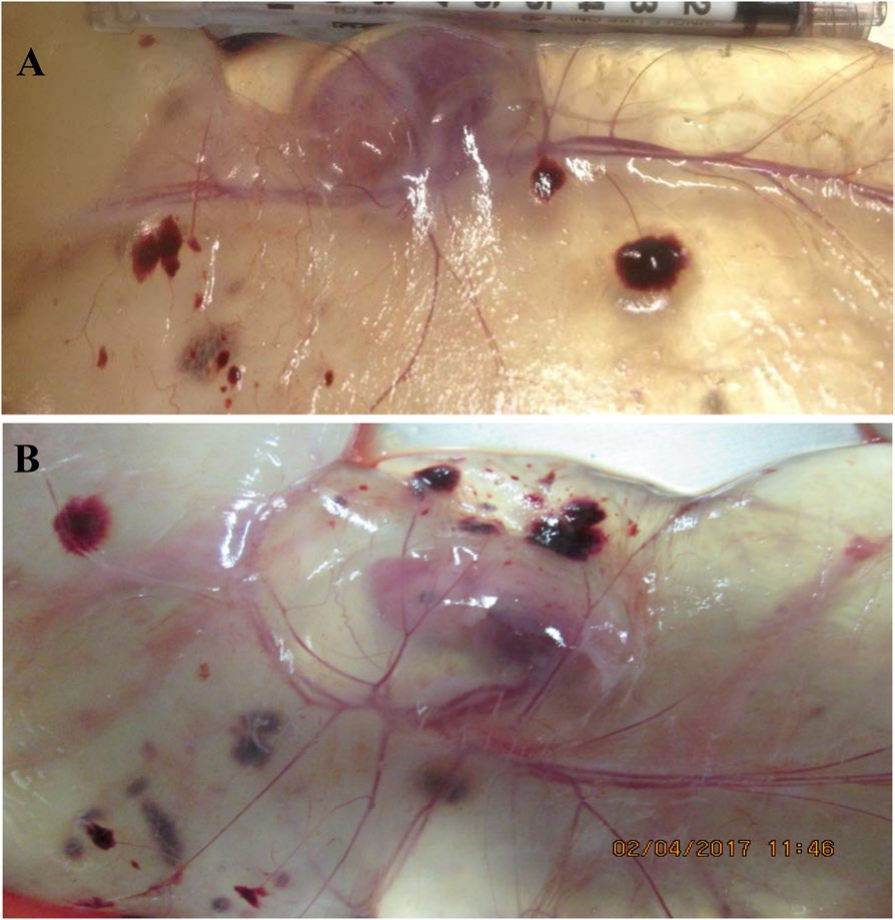
Placental blood vessels in the allantois on d 30 of gestation in gilts supplemented with either 0 (control) or 0.4% *L*-arginine. Placental blood vessels are shown in the allantois of gilts without *L*-arginine supplementation (control; Panel A) and gilts receiving dietary supplementation with 0.4% *L*-arginine (Panel B)

Angiogenesis of placentae was determined by counting the number of their blood vessels and measuring their diameter. The number of blood vessels per cm^2^ and their diameter in the placentae of the 0.4% Arg-supplemented group were 25.9% and 17.0% greater (*P* < 0.05), respectively, compared with control gilts (Table 2). As shown in Fig. 1, the blood vessels of the allantois of placentae of conceptuses in 0.4% Arg-supplemented gilts (Panel B) were more developed and more abundant than those in the allantois of placentae of conceptuses in control gilts (Panel A).

### Placental transport of ^3^H_2_O and ^14^C-AAs

Water and AAs were readily transported across the placental tissue from the “mucosal” side to the “serosal” side of Ussing chambers at a constant rate during the 15-min period of measurement (Table 7). Compared with control gilts, dietary supplementation with 0.4% Arg increased (*P* < 0.01) the rates of net transport of water (33.9%), Arg (78.4%), *L*-glutamine (89.9%), and glycine (89.6%) by placentae (Table 7).

**Table 7:** Rates of the net transport of water and amino acids by placentae from gilts fed diets supplemented with 0 (control) or 0.4% *L*-arginine (Arg) between d 14 and d 30 of gestation

Time, min	Net transport of water, nL/mg tissue/min		Net transport of Arg, pmol/mg tissue/min		Net transport of Gln, pmol/mg tissue/min		Net transport of Gly, pmol/mg tissue/min	
	Control	0.4% Arg	Control	0.4% Arg	Control	0.4% Arg	Control	0.4% Arg
5	356 ± 14	475 ± 20^*^	32.1 ± 2.2	58.8 ± 3.6^*^	33.4 ± 2.5	64.1 ± 4.2^*^	65.9 ± 3.3	123.8 ± 6.6^*^
10	352 ± 17	473 ± 21^*^	32.5 ± 2.0	57.3 ± 3.2^*^	33.0 ± 2.2	62.8 ± 3.6^*^	65.4 ± 3.8	125.2 ± 6.2^*^
15	358 ± 15	479 ± 22^*^	31.9 ± 1.7	56.1 ± 3.9^*^	33.7 ± 2.4	63.2 ± 4.0^*^	65.6 ± 3.6	124.4 ± 6.9^*^

Values are means ± SEM, *n* = 10 gilts/treatment group. On d 30 of gestation, pieces of placental tissue (1 cm^2^) were mounted onto Ussing chambers, followed by the addition of either ^3^H_2_O (20 µL), 0.2 mmol/L *L*-arginine (Arg) plus *L*-[U-^14^C]Arg, 0.5 mmol/L *L*-glutamine (Gln) plus *L*-[U-^14^C]Gln, or 1 mmol/L glycine (Gly) plus [U-^14^C]Gly to the “mucosal” side of the Ussing chambers for their net transport into the “serosal side” of the Ussing chambers

^*^P < 0.01 vs. the corresponding control group

### Expression of angiogenic factors and AQPs in the placenta

qPCR was performed on placental tissue at d 30 of gestation from gilts supplemented with either 0 (control) or 0.4% Arg from d 14 to d 30 of gestation to analyze the mRNA expression of key factors associated with angiogenesis and water transport. Results are summarized in Table 8. *VEGFA120* and *VEGFA164* are the two isoforms of *VEGF*. The placental mRNA level for *VEGFA120* was 117% greater (*P* < 0.05) in the 0.4% Arg-supplemented gilts, compared to the control group, but the placental mRNA level for *VEGFA164* did not differ (*P* > 0.05) between these two groups of gilts. Placental mRNAs for *VEGFR1* and *VEGFR2*, as well as *PGF* and *GCH1* were 445%, 373%, 197%, and 126% more abundant (*P* < 0.01), respectively, in the 0.4% Arg-supplemented gilts than those in control gilts. By contrast, dietary supplementation with 0.4% Arg did not affect (*P* > 0.05) placental mRNA levels for *NOS3* or *FGF2*.

**Table 8:** Relative expression of mRNAs for angiogenic factors and AQPs in the placentae of gilts fed a diet supplemented with 0.4% *L*-arginine (Arg) versus 0 Arg between d 14 and d 30 of gestation

Gene	Fold change	*P*-value
*VEGFA120*	1.17	0.031
*VEGFA164*	0.99	0.948
*VEGFR1*	4.45	0.008
*VEGFR2*	3.73	< 0.001
*NOS3*	1.14	0.145
*PGF*	1.97	< 0.001
*GCH1*	1.26	0.001
*FGF2*	0.96	0.144
*AQP1*	2.80	0.002
*AQP2*	1.09	0.763
*AQP3*	1.37	0.046
*AQP4*	1.05	0.148
*AQP5*	1.72	0.047
*AQP8*	1.65	0.004
*AQP9*	1.27	0.020
*AQP11*	1.14	0.806

Values are the relative expression of genes in the placentae of gilts supplemented with 0.4% *L*-arginine (9 gilts), compared to gilts supplemented with 0 *L*-arginine (control; 7 gilts) between d 14 and d 30 of gestation. The abundances of mRNAs for the genes were measured by qPCR using SYBR Green

AQPs 1, 2, 3, 4, 5, 8, 9, and 11 were expressed in the placentae of gilts on d 30 of gestation (Table 8). mRNA for *AQP10* was not detected in the placentae from control or Arg-supplemented gilts. Dietary supplementation with 0.4% Arg enhanced (*P* < 0.05) mRNA levels for *AQP1* (280%), *AQP3* (137%), *AQP5* (172%), *AQP8* (165%), and *AQP9* (127%) in the porcine placentae (Table 7). There was no difference (*P* > 0.05) in placental mRNA levels for *AQP2*, *AQP4*, and *AQP11* between the control and 0.4% Arg-supplemented gilts.

## Discussion

Because swine experience high rates of embryonic mortality during early gestation, a management practice to ameliorate such loss would be highly beneficial to both the swine industry and researchers [3, 4, 6, 59, 60]. A corn- and soybean meal-based diet containing 12% crude protein is considered optimal to provide most AAs and prevent hyperammonemia (a major factor contributing to embryonic death) in gestating pigs [8]. However, a gestation diet containing 12% crude protein does not meet dietary requirements for Arg [5, 12]. Thus, supplementing this deficient AA to the maternal diet is an effective way to enhance the growth and development of the conceptus without any detrimental effects associated with increasing dietary crude protein intake [5–7, 26, 61, 62]. Most embryonic loss in pigs occurs before d 30 of gestation, making this time period an appropriate target for improvement in the reproductive performance of gilts and sows [2–4, 24]. However, Li et al. [29] discovered that dietary supplementation with 0.8% Arg between d 0 to d 25 decreased the number of CL and, accordingly, litter size, and concentrations of progesterone in maternal plasma due to excessive NO generation. In a subsequent study, Li et al. [24] found that dietary supplementation with 0.4% and 0.8% Arg to gilts from d 14 to d 25 of gestation enhanced litter size by 2 conceptuses, as well as allantoic and amniotic fluid volumes, when compared with control gilts. Of note, dietary supplementation with 1.075% Arg to sows (parity ≥ 2; an average of approximately 4) from d 1 to d 30 of gestation increased the number of piglets born alive per litter by 1.63 [61]. The dose of Arg supplementation is important to prevent an imbalance among basic AAs in diets [62, 63]. Therefore, total dietary Arg should be less than 2%, so that the ratio of Arg to lysine does not exceed 3 to prevent competition for transport into cells between these two basic AAs [63]. We used a supplemental dose of 0.4% Arg in the present study, because this amount was determined to be sufficient for enhancing the survival and development of conceptuses in gestating gilts [24].

NO is a potent vasodilator and also stimulates placental angiogenesis [13, 14, 22]. Specifically, NO enhances blood flow through inducing the dilation of the blood vessels and increasing vascular density via a cGMP-dependent mechanism [19, 22]. The placental vasculature is responsible for the delivery of nutrients and gases for exchange across the utero-placental interface between mother and fetus, as well as for the removal of fetal metabolic wastes [64, 65]. Our results suggest that Arg increases placental angiogenesis by increasing the expression of genes for angiogenic factors, such as *VEGFA120*, *VEGFR1*, *VEGFR2*, *PGF*, and *GCH1* (Table 8). VEGFA is considered as the conventional form of VEGF and acts on endothelial cells to induce their migration and proliferation along with increasing the endothelial production of NO [13]. *VEGFA120* and *VEGFA164* are splice variants of *VEGFA* expressed in the porcine placenta to increase vascular permeability [65]. VEGFA binds to the VEGF receptors 1 and 2, thereby exerting its physiological function [66, 67]. PGF is also part of the VEGF family that acts in synergy with VEGF to promote angiogenesis [67]. As noted previously, eNOS converts Arg to NO in endothelial cells [19] and GTP-CH1 is a rate-controlling enzyme in the production of BH_4_ [51, 52], which is an essential cofactor of all NOS isoforms for NO synthesis [17]. As reported for endothelial cells in both normal and diabetic rats [51, 52], Arg increases the synthesis and bioavailability of BH_4_, thereby increasing the generation of NO by the porcine placentae (Table 6). In addition to NO, Arg supplementation augmented placental ODC activity as well as the availability of both Arg and proline (the major sources of ornithine in the porcine placenta [45]) due to enhanced AA transport (Table 7)] for syntheses of NO and polyamines (Table 6) that also stimulate angiogenesis [21, 68, 69]. Elevated expression of these angiogenic factors increases angiogenic activity (including the proliferation of endothelial cells) in the placentae, resulting in a more highly developed placental vasculature (Table 2 and Fig. 1). Therefore, more water and AAs can be transported across the placenta (Table 7) for use by the embryo/fetus and for storage in both allantoic and amniotic fluids (Tables 2 and 4).

The 12 AQPs (AQPs 1–12) expressed in the female reproductive tract can be classified into three different subgroups [33, 70, 71]. AQPs 1, 2, 4, 5, 6 and 8 are classical aquaporins that are highly selective for water transport. AQPs 3, 7, 9 and 10 are aquaglyceroporins that transport urea, glycerol, and other small solutes in addition to water. AQPs 11 and 12 are superaquaporins. As reported by Zhu et al. [33] for porcine placentae on d 25 of gestation, the placentae of gilts expressed *AQP*1, *AQP*2, *AQP*3, *AQP*4, *AQP*5, *AQP*8, *AQP*9, and *AQP*11, but not *AQP10*, on d 30 of gestation (Table 8). Most recently, McLendon et al. [38] localized AQP 1, 5, 8, and 9 proteins to specific cell types within both the endometrium and placenta, suggesting that pigs can use AQP1, AQP5, AQP8, and AQP9 to transport water from the endometrial bloodstream to the allantoic bloodstream and allantoic fluid. Much evidence shows that AQPs are essential for maintaining the accumulation and reabsorption of allantoic and amniotic fluids for optimal embryonic growth [37]. Of note, dietary supplementation with Arg enhanced the expression of genes for *AQP*1, *AQP*3, *AQP*5, *AQP*8, and *AQP*9 in the placenta (Table 8) and functionally the placental transport of water (Table 7). This finding is consistent with the report that Arg enhanced the expression of AQP3 in porcine trophectoderm cells [36] and our additional observation that allantoic and amniotic fluid volumes in fetal pigs were much greater in gilts receiving dietary supplementation with 0.4% Arg as compared with control gilts (Table 2). There is clear evidence that volumes of these fetal fluids are positively correlated with embryonic growth and survival in mammals, including pigs [30, 31, 59].

**Fig. 2:**
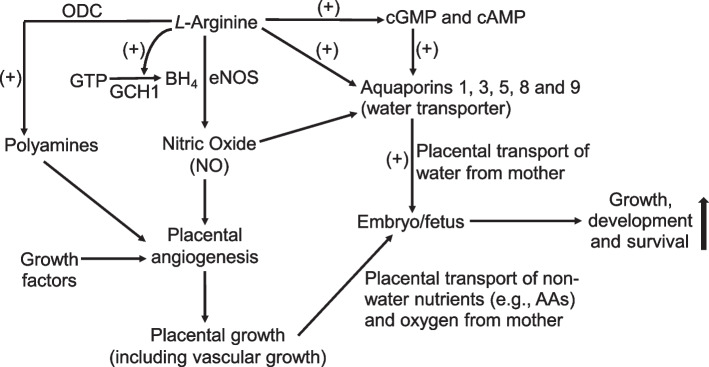
Proposed mechanisms responsible for beneficial effects of dietary *L*-arginine supplementation in improving embryonic/fetal growth and survival in gestating swine. *L*-Arginine stimulates the synthesis of tetrahydrobiopterin [BH_4_, a required co-factor for nitric oxide (NO) synthase)] from GTP via the GTP cyclohydrolase-I (GCH1) pathway, thereby augmenting NO production by placental tissue. *L*-Arginine also increases the activity of ornithine decarboxylase (a key enzyme for the synthesis of polyamines). Both NO and polyamines, as well as growth factors (such as placental growth factor, vascular endothelial growth factor A120, and vascular endothelial growth factor receptors 1 and 2) promote placental angiogenesis and growth (including vascular growth) to increase rates of transfer of non-water nutrients [including amino acids (AAs)] and oxygen across the placenta from mother to embryo/fetus. In addition, *L*-arginine elevates the concentrations of both cGMP and cAMP in the placenta to increase the expression of aquaporins (AQPs) to promote the placental transport of water from mother to embryo/fetus. Ultimately, the coordinate actions of *L*-arginine result in improvements in the growth and survival of embryos/fetuses

As reported for endothelial cells and skeletal muscle of Arg-supplemented rats [69], dietary Arg supplementation increased the concentrations of both cAMP and cGMP in porcine placentae (Table 6). Some AQPs (e.g., AQPs 1 and 5) are cGMP-gated transmembrane channels [70, 71]. In addition, AQPs are activated by cAMP-dependent protein kinase A [32, 37]. Thus, cGMP and cAMP cell signaling can up-regulate water transport across the cell membrane. In support of this view, AQP3 expression was enhanced by Forskolin (a cell-permeable activator of adenylate cyclase) but inhibited by H-89 (an inhibitor of cAMP-dependent protein kinase) in porcine conceptus trophectoderm cells [36]. Furthermore, the addition of a membrane-permeable cGMP analog (i.e., para-chlorophenylthio-cGMP) to culture medium stimulated water transport across the human pigmented retinal epithelium [71] and porcine conceptus trophectoderm cells [36]. Likewise, addition of diethylenetriamine-nitric oxide adduct (DETA-NO; an NO donor; 15 µmol/L) to the “mucosal side” of Ussing chambers rapidly enhanced water transport by placentae from gilts on d 60 of gestation (i.e., 36% and 86% at 2 and 10 min, respectively, compared with the absence of DETA-NO) [72]. Conversely, inhibition of NO synthesis reduced water transport by porcine placental cells [36]. Because NO stimulates the production of cGMP from GTP by guanylate cyclase in cells [37], dietary supplementation with 0.4% Arg enhanced the concentration of cGMP in porcine placentae by 25% (Table 6). Similarly, increasing the extracellular concentration of Arg from 0.1 to 0.25 mmol/L augmented the concentration of cGMP in porcine trophectoderm cells by 38% [72]. These results support the previous conclusion from in vitro studies [36] that the NO-cGMP and cAMP-dependent pathways play an important role in promoting water transport by the placentae of Arg-supplemented gilts to increase the volumes of allantoic and amniotic fluids of the conceptuses (Fig. 2). Arg is truly a functional AA for successful pregnancy outcomes in mammals (including swine) and must be included adequately in their diets [73, 74].

## Conclusions

Results of the present study revealed new insights into the mechanisms whereby dietary supplementation with 0.4% Arg to gilts between d 14 and d 30 of gestation enhances embryonic survival, as well as the volumes of allantoic and amniotic fluids in the conceptuses. In addition, Arg supplementation increased the syntheses of NO and polyamines by placentae, the expression of angiogenic factors and angiogenesis in placentae (as indicated by increases in the number of placental blood vessels and their diameters), placental growth and AQP expression, and the placental transport of water and AAs. These results advance the understanding of mechanisms whereby dietary Arg supplementation beneficially improves embryonic/fetal growth and survival. Our findings have important nutritional implications for increasing reproductive performance in swine and other mammalian species.

## Data Availability

All data generated or analyzed during this study are available from the corresponding author upon reasonable request.

## Abbreviations

AA: Amino acid
AQP: Aquaporin
BH_4_: Tetrahydrobiopterin
CL: Corpora luteum
cNOS: Constitutive nitric oxide synthase
eNOS (NOS3): Endothelial nitric oxide synthase
FGF2: Fibroblast growth factor 2
GTP-CH1 (or GCH1): GTP cyclohydrolase-1
HPLC: High-performance liquid chromatography
iNOS: Inducible nitric oxide synthase
NO: Nitric oxide
NOx: Stable oxidation products of NO (nitrite plus nitrate)
ODC: Ornithine decarboxylase
PGF: Placental growth factor
TUBA1B: Tubulin α 1b
VEGFA: Vascular endothelial growth factor A
VEGFR: Vascular endothelial growth factor receptor
